# Progressive Pseudorheumatoid Dysplasia Misdiagnosed as Juvenile Idiopathic Arthritis: Advanced Joint Destruction

**DOI:** 10.7759/cureus.92559

**Published:** 2025-09-17

**Authors:** Wassima Ridah, Soukaina Zaher, Ahlam Ajerouassi, Kawtar Nassar, Saadia Janani

**Affiliations:** 1 Department of Rheumatology, Ibn Rochd University Hospital Center, Faculty of Medicine and Pharmacy of Casablanca, Casablanca, MAR

**Keywords:** arthropathy, genetic skeletal disorder, joint prosthesis loosening, juvenile idiopathic arthritis, misdiagnosis, progressive pseudorheumatoid dysplasia, wisp3 protein

## Abstract

Progressive pseudorheumatoid dysplasia (PPRD) is a rare autosomal recessive arthropathy often misdiagnosed as juvenile idiopathic arthritis (JIA). We present the case of a 52-year-old woman with polyarthralgia since age 7, initially diagnosed with seronegative polyarticular JIA and treated with methotrexate at doses ranging from 10 to 15 mg weekly for over two decades, despite the absence of clinical improvement. The rationale for prolonged use was the persistence of joint symptoms under the initial diagnosis of JIA, leading clinicians to continue disease-modifying therapy. Clinical examination revealed joint deformities, limited range of motion, short stature, and limb length discrepancy. Radiographs showed characteristic PPRD features, including flattening of the metacarpophalangeal joints, brachymetatarsia, vertebral fractures, and lumbar osteopenia. Pelvic imaging demonstrated bilateral hip prostheses with loosening of the right prosthesis, superior migration of the femoral component, and screw fracture. Laboratory tests revealed normal inflammatory markers and negative rheumatoid factor and anti-cyclic citrullinated peptide (anti-CCP) antibodies. This case highlights the diagnostic challenges of PPRD and the potential for irreversible joint destruction when diagnosis is delayed. Clinicians should suspect PPRD in early onset polyarthritis with non-inflammatory joint involvement and negative immunologic tests to avoid unnecessary immunosuppressive therapy and initiate timely supportive management.

## Introduction

Progressive pseudorheumatoid dysplasia (PPRD) is a rare autosomal recessive arthropathy characterized by abnormal cartilage lysis affecting multiple joints [1]. The earliest descriptions of conditions resembling PPRD date back to Kahn et al. in 1970, who reported cases under the term “chondrodysplastic rheumatism” [2]. In 1972, Laplane et al. described familial “syndesmodysplastic dwarfism,” further highlighting the features of a hereditary skeletal dysplasia with progressive joint involvement [3]. In 1980, Spranger et al. consolidated these earlier observations and established the disorder as a distinct clinical entity, introducing the term “progressive pseudorheumatoid dysplasia” [2]. PPRD is caused by autosomal recessive mutations in WISP3 (Wnt1-inducible signaling pathway protein 3), which encodes a cysteine-rich signaling protein essential for cartilage metabolism and extracellular matrix homeostasis. Functional loss of WISP3 compromises cartilage integrity, leading to abnormal skeletal development and progressive, non-inflammatory joint degeneration [4,5]. The estimated prevalence of PPRD is 1 in 1,000,000, with the majority of cases concentrated along the eastern Mediterranean rim, in the Middle East, and in the Maghreb. However, there have been isolated instances of individuals of European descent affected by the disease [6,7]. The symptoms of the disease generally appear between the ages of 3 and 8, presenting with pain, swelling, and stiffness in multiple joints, particularly in the hands [8]. Due to its similarities with juvenile idiopathic arthritis (JIA), PPRD is frequently misdiagnosed, resulting in inappropriate treatment and prolonged suffering [9]. Although it presents with early onset and polyarticular involvement, PPRD differs by the absence of systemic inflammation, persistently negative immunologic markers, and the presence of characteristic radiographic changes, which help distinguish it from JIA [5]. Early recognition of this condition is crucial for effective management and improved patient outcomes. This report presents the case of a 52-year-old woman with PPRD, initially misdiagnosed as seronegative polyarticular JIA, presenting with advanced joint destruction and prosthesis-related complications.

## Case presentation

**Figure 1:**
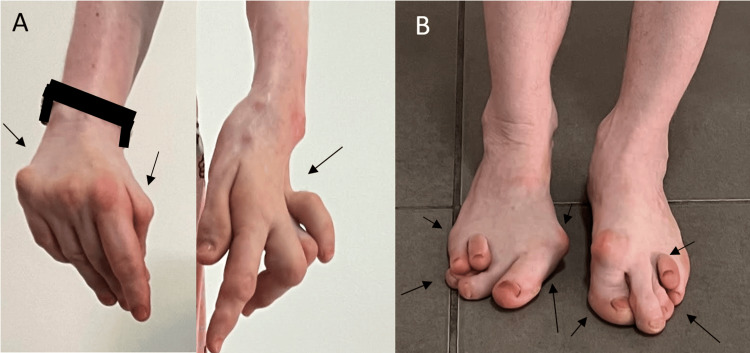
Clinical findings. A. Bilateral hand amyotrophy with a Z-thumb. Boutonnière deformity in the third, fourth, and fifth left fingers. B. Bilateral hallux valgus and clawed toes.

**Figure 2:**
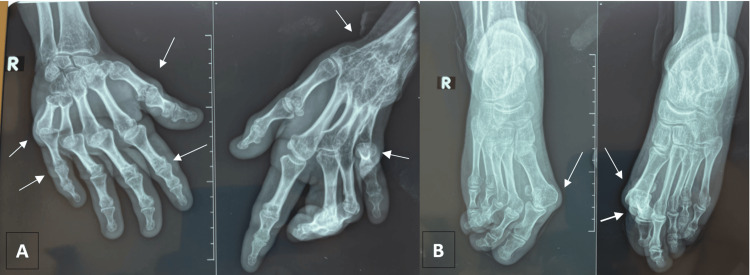
X-ray of the hands and feet. A. Hands X-ray showing flattening and epi-metaphyseal enlargement of the metacarpophalangeal and proximal interphalangeal joints and ankylosis of the left radiocarpal, intercarpal, and carpometacarpal joints. B. Feet X-ray revealing bilateral subluxation of the first metatarsophalangeal joint, along with subluxation of the second and third metatarsophalangeal joints on the right foot, shortening of the left first intermediate phalanx, and brachymetatarsia of the fourth metatarsal.

**Figure 3:**
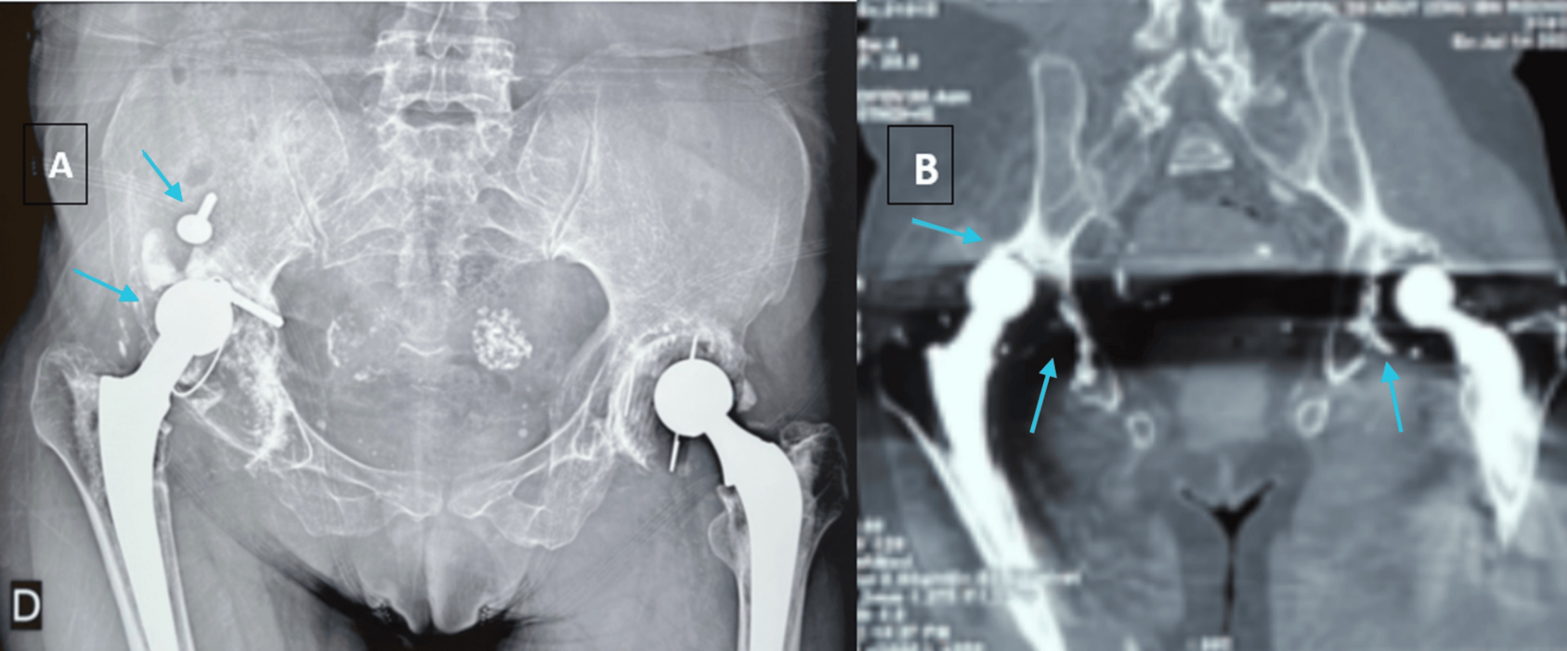
Pelvic radiograph (A) and coronal CT scan (B) demonstrating loosening of the right total hip prosthesis. A. Anteroposterior pelvic radiograph showing bilateral total hip prostheses. The right prosthesis demonstrates clear signs of loosening, with superior migration of the femoral component and fracture of one of the fixation screws, associated with peri-prosthetic bone changes. B. Coronal CT reconstruction showing mechanical loosening of the right prosthesis, with marked peri-acetabular bone loss and displacement of the femoral component.

**Figure 4:**
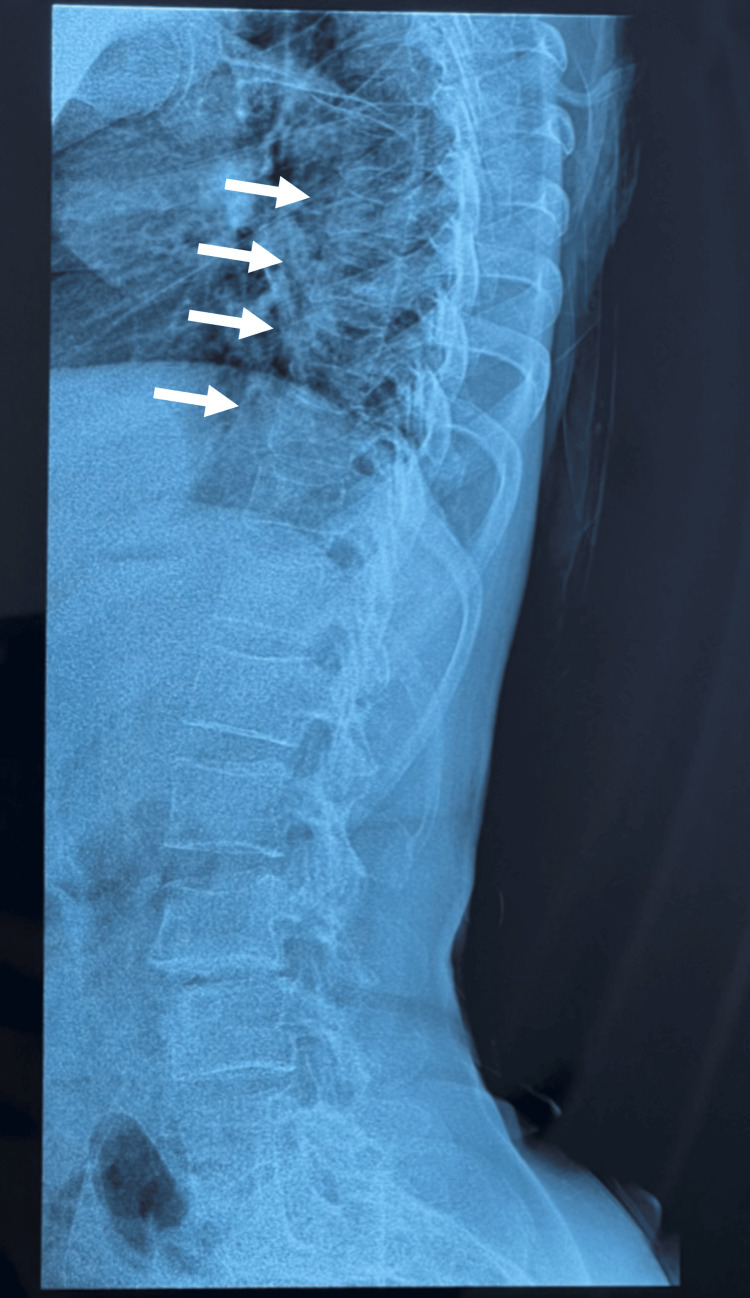
Lateral thoracic and lumbar spine X-ray demonstrating multiple vertebral fractures (notably D7, D8, D9, and D10). Degenerative changes are also visible, including reduced intervertebral disc height at several thoracic and lumbar levels and irregularity of the vertebral endplates.

A 52-year-old woman with a medical history of high blood pressure, diabetes, vitiligo, premature menopause, and two total hip prostheses was admitted to the rheumatology department. She is not known to be born from a consanguineous union, and no similar cases were reported among her siblings. The patient has presented with polyarthralgia and non-synovial swelling affecting the metacarpophalangeal joints, proximal interphalangeal joints, wrists, elbows, shoulders, knees, and ankles since the age of 7. She was initially diagnosed with seronegative polyarticular JIA, which subsequently evolved into seronegative rheumatoid arthritis. She has received methotrexate at doses ranging from 10 to 15 mg weekly for over two decades despite the absence of clinical improvement. The rationale for prolonged use was the persistence of joint symptoms under the initial diagnosis of JIA, leading clinicians to continue disease-modifying therapy in the hope of achieving delayed efficacy and preventing further joint damage. Upon physical examination, the patient was observed to have a shorter stature than average (height 1.49 m) and a weight of 54 kg, corresponding to a BMI of 24.3 kg/m². The patient presented with joint pain, non-synovial swelling, limited range of movement, amyotrophy of the hands (Figure 1A) , deltoids, and right thigh, and joint deformities affecting the hands, elbows, and feet (Figure 1B). The articular involvement was symmetric, affecting both upper and lower limb joints. The deformities also included shortening of the left forearm and the right lower limb due to the removal of the hip prosthesis. The erythrocyte sedimentation rate and C-reactive protein levels were within the normal range. The results of the rheumatoid factor and anti-cyclic citrullinated peptide (anti-CCP) tests were negative. X-ray imaging of the hands revealed bilateral flattening and epi-metaphyseal widening of the proximal metacarpophalangeal and interphalangeal joints, accompanied by ankylosis of the left radiocarpal, intercarpal, and carpometacarpal joints (Figure 2A). An X-ray of the feet revealed deformities, including subluxation of the metatarsophalangeal joints and brachymetatarsia (Figure 2B). A pelvic radiograph (Figure 3A) showed bilateral total hip prostheses, with loosening of the right prosthesis, superior migration of the femoral component, and fracture of one of the fixation screws. Coronal CT imaging (Figure 3B) also demonstrated mechanical loosening with marked peri-acetabular bone loss and displacement of the femoral component. Radiological findings on the spine revealed the presence of vertebral fractures (Figure 4), while the DXA scan identified osteopenia in the lumbar spine, with a T-score of -2.1. Unfortunately, the patient did not have the financial means to undergo genetic testing to confirm WISP3 mutations. Nevertheless, genetic confirmation should be recommended whenever feasible, as it provides a definitive diagnosis and enables appropriate genetic counseling. Given the combination of characteristic clinical, biological, and radiological features, together with the absence of response to disease-modifying antirheumatic drugs, the diagnosis of PPRD was considered highly reliable despite the lack of molecular confirmation. She was treated with analgesics and physical rehabilitation, as well as bisphosphonates, which are indicated in cases of osteopenia with vertebral fractures. In regard to the discomfort experienced in the right hip, a re-evaluation of the arthroplasty is warranted.

## Discussion

PPRD is an autosomal recessive genetic skeletal disorder classified in groups 13 and 31 in the 2011 Classification of Genetic Skeletal Disorders [10]. It is caused by mutations in the Wnt1-inducible signaling pathway protein 3 (WISP3) gene, which encodes secreted cysteine-rich proteins involved in regulating cell growth and differentiation [11].

Children with PPRD are usually healthy at birth and develop normally during early childhood. Symptoms typically begin between the ages of 3 and 8, though onset can be delayed until adolescence or, in some cases, present as early as the first year of life [12]. Early manifestations often include gait abnormalities, fatigue, and symmetrical joint stiffness - particularly in the hips - accompanied by enlargement of the interphalangeal joints of the hands. Pain may be absent or mild initially, becoming more noticeable over time but often remaining disproportionate to the degree of joint damage. Ankylosis can occur in multiple joints, including the shoulders, wrists, elbows, ankles, and metatarsophalangeal joints, while temporomandibular involvement is uncommon [13].

As the disease progresses, skeletal changes become more pronounced, leading to kyphoscoliosis, short stature (below the third percentile), joint contractures, progressive hip degeneration, and camptodactyly. Certain genotypes may be associated with additional features such as humeral involvement and upper limb shortening. While height is generally normal in infancy, growth velocity decreases in most patients, and many present with short stature at diagnosis [5,12,14].

Laboratory tests are usually normal, with no elevation of inflammatory markers, and rheumatoid factor and antinuclear antibody tests are negative [1,15]. Imaging plays a central role in diagnosis. Early radiographs may show mild metaphyseal widening of the interphalangeal joints, which evolves toward joint space narrowing and ankylosis. The spine may present defective ossification and platyspondyly, contributing to kyphoscoliosis [16]. Hip involvement is characterized by enlarged, flattened femoral heads with acetabular overgrowth and a distinct lip [12]. Flattened joint surfaces may also be observed in the knees, and the ankle can show an enlarged, irregular os trigonum [6,17].

Our patient’s presentation combined these classical features with advanced joint destruction. Imaging demonstrated epi-metaphyseal widening and ankylosis in the hands, brachymetatarsia and metatarsophalangeal subluxations in the feet, vertebral fractures on spinal radiographs, and lumbar osteopenia on bone densitometry. The most striking finding was pelvic imaging, which revealed bilateral hip prostheses with loosening of the right prosthesis, superior migration of the femoral component, fracture of a fixation screw, and peri-acetabular bone loss. Such severe hip damage, requiring arthroplasty and complicated by prosthetic loosening, is rarely described in the literature and illustrates the long-term consequences of delayed diagnosis and prolonged exposure to ineffective treatment, in this case methotrexate for over two decades.

In this case, the primary differential diagnosis considered was JIA, given the early onset of polyarticular symptoms. However, the absence of systemic inflammatory manifestations, persistently negative immunologic markers, and the lack of clinical response to long-term methotrexate therapy argued strongly against JIA. Osteoarthritis was also considered but was excluded due to the patient’s age of onset, as well as the distribution and symmetry of joint involvement. Other skeletal dysplasias, such as spondyloepiphyseal dysplasia or metaphyseal chondrodysplasia, were less compatible with the observed radiographic pattern, which was highly suggestive of PPRD.

Genetic testing offers definitive confirmation of PPRD and is especially important for preventing misdiagnosis, guiding accurate genetic counseling, and detecting potential familial involvement. Although the absence of molecular testing in our patient represents a limitation, the diagnosis remains highly reliable in light of the characteristic clinical and radiological features.

Management of PPRD is essentially supportive, aiming to control pain, maintain mobility, and prevent complications. Physical rehabilitation plays a key role, while immunosuppressive agents such as methotrexate have shown no benefit in modifying disease progression [5]. In advanced cases, surgical interventions including joint replacement may be required, although underlying bone fragility and deformities can complicate outcomes. Early and accurate recognition is therefore crucial to avoid unnecessary treatments and implement targeted supportive measures.

## Conclusions

This case illustrates the diagnostic challenges associated with PPRD, a rare genetic skeletal disorder that is often misidentified as JIA. Our patient was treated for over two decades with immunosuppressants under a misdiagnosis, highlighting the consequences of delayed recognition. Clinicians should consider PPRD in the differential diagnosis of early onset polyarthritis, particularly in the presence of non-inflammatory joint involvement, negative immunologic markers, and characteristic radiological features. Genetic confirmation should be pursued whenever feasible. Early recognition is essential not only to implement appropriate supportive care but also to avoid unnecessary and ineffective immunosuppressive therapy, thereby preserving function and improving quality of life.
